# Entropy-Based Greedy Algorithm for Decision Trees Using Hypotheses

**DOI:** 10.3390/e23070808

**Published:** 2021-06-25

**Authors:** Mohammad Azad, Igor Chikalov, Shahid Hussain, Mikhail Moshkov

**Affiliations:** 1 Department of Computer Science, College of Computer and Information Sciences, Jouf University, Sakaka 72441, Saudi Arabia; mmazad@ju.edu.sa; 2 Intel Corporation, 5000 W Chandler Blvd, Chandler, AZ 85226, USA; igor.chikalov@gmail.com; 3 Computer Science Program, Dhanani School of Science and Engineering, Habib University, Karachi 75290, Pakistan; hussain.shahid@gmail.com; 4 Computer, Electrical and Mathematical Sciences & Engineering Division, King Abdullah University of Science and Technology (KAUST), Thuwal 23955-6900, Saudi Arabia

**Keywords:** decision tree, hypothesis, greedy algorithm, entropy

## Abstract

In this paper, we consider decision trees that use both conventional queries based on one attribute each and queries based on hypotheses of values of all attributes. Such decision trees are similar to those studied in exact learning, where membership and equivalence queries are allowed. We present greedy algorithm based on entropy for the construction of the above decision trees and discuss the results of computer experiments on various data sets and randomly generated Boolean functions.

## 1. Introduction

Decision trees are well known as means for knowledge representation, as classifiers, and as algorithms to solve various problems of combinatorial optimization, computational geometry, etc. [1,2,3].

Conventional decision trees have been studied in different theories, in particular, in rough set theory initiated by Pawlak [4,5,6] and in test theory initiated by Chegis and Yablonskii [7]. These trees use simple queries based on one attribute each.

In contrast to these theories, exact learning initiated by Angluin [8,9] studied not only membership queries that correspond to attributes from rough set theory and test theory, but also so-called equivalence queries. Relations between exact learning and probably approximately correct (PAC) learning proposed by Valiant [10] were discussed in [8].

In this paper, we add the notion of a hypothesis to the model that has been considered in rough set theory as well in test theory. This model allows us to use an analog of equivalence queries.

Let *T* be a decision table with *n* conditional attributes $f_{1},\ldots ,f_{n}$ having values from the set ω={0,1,2,…} in which rows are pairwise different and each row is labeled with a decision from ω. For a given row of *T*, we should recognize the decision attached to this row. To this end, we can use decision trees based on two types of queries. We can ask about the value of an attribute $f_{i}\in \left\{f_{1},\ldots ,f_{n}\right\}$ on the given row. We will obtain an answer of the kind $f_{i}=\delta$, where δ is the number in the intersection of the given row and the column $f_{i}$. We can also ask if hypothesis $f_{1}=\delta_{1},\ldots ,f_{n}=\delta_{n}$ is true, where $\delta_{1},\ldots ,\delta_{n}$ are numbers from the columns $f_{1},\ldots ,f_{n}$, respectively. Either this hypothesis will be confirmed or we will obtain a counterexample in the form $f_{i}=\sigma$, where $f_{i}\in \left\{f_{1},\ldots ,f_{n}\right\}$ and σ is a number from the column $f_{i}$ different from $\delta_{i}$. The considered hypothesis is called proper if $\left(\delta_{1},\ldots ,\delta_{n}\right)$ is a row of the table *T*.

In this paper, we consider two cost functions that characterize the time and space complexity of decision trees. We consider the depth of a decision tree as its time complexity, which is equal to the maximum number of queries in a path from the root to a terminal node of the tree. As the space complexity of a decision tree, we consider the number of its realizable, relative to *T*, nodes. A node is called realizable relative to *T* if for a row of *T* and some choice of counterexamples the computation in the tree passes through this node.

Decision trees using hypotheses can be essentially more efficient than the decision trees using only attributes. Let us consider an example, the problem of computation of the conjunction $x_{1}\wedge \cdots \wedge x_{n}$. The minimum depth of a decision tree solving this problem using the attributes $x_{1},\ldots ,x_{n}$ is equal to *n*. The minimum number of realizable nodes in such decision trees is equal to 2n+1. However, the minimum depth of a decision tree solving this problem using proper hypotheses is equal to 1: it is enough to ask only about the hypothesis $x_{1}=1,\ldots ,x_{n}=1$. If it is true, then the considered conjunction is equal to 1. Otherwise, it is equal to 0. The obtained decision tree contains n+2 realizable nodes.

In this paper, we consider the following five types of decision trees:

1. Decision trees that use only attributes.
2. Decision trees that use only hypotheses.
3. Decision trees that use both attributes and hypotheses.
4. Decision trees that use only proper hypotheses.
5. Decision trees that use both attributes and proper hypotheses.

There are different ways to construct conventional decision trees, including algorithms that can construct optimal decision trees for medium-sized decision tables [11,12,13,14,15]. In particular, in [16,17], we proposed dynamic programming algorithms for the minimization of the depth and number of realizable nodes in decision trees with hypotheses. However, the most common way is to use greedy algorithms [1,18].

In this paper, we propose a greedy algorithm based on entropy that, for a given decision table and type of decision trees, constructs a decision tree of the considered type for this table. The goal of this paper is to understand which type of decision tree should be chosen if we would like to minimize the depth and which type should be chosen if we would like to minimize the number of realizable nodes. To this end, we compare the parameters of the constructed decision trees of five types for 10 decision tables from the UCI ML Repository [19]. We do the same for randomly generated Boolean functions with *n* variables, where n=3,…,6. From the obtained experimental results, it follows that we should choose decision trees of the type 3 if we would like to minimize the depth and decision trees of the type 1 if we would like to minimize the number of realizable nodes.

The main contributions of the paper are (i) the design of the extended entropy-based greedy algorithm that can work with five types of decision trees and (ii) the understanding of which type of decision trees should be chosen when we would like to minimize the depth or the number of realizable nodes.

The rest of the paper is organized as follows. In Section 2 and Section 3, we consider the main notions and in Section 4, the greedy algorithm for the decision tree construction. Section 5 contains the results of the computer experiments and Section 6, short conclusions.

## 2. Decision Tables

A decision table is a rectangular table *T* with n≥1 columns filled with numbers from the set ω={0,1,2,…} of non-negative integers. Columns of this table are labeled with the conditional attributes $f_{1},\ldots ,f_{n}$. Rows of the table are pairwise different. Each row is labeled with a number from ω that is interpreted as a decision. Rows of the table are interpreted as tuples of values of the conditional attributes.

A decision table *T* is called empty if it has no rows. The table *T* is called degenerate if it is empty or all rows of *T* are labeled with the same decision.

We denote $F\left(T\right)=\left\{f_{1},\ldots ,f_{n}\right\}$ and denote by D(T) the set of decisions attached to the rows of *T*. For any conditional attribute $f_{i}\in F\left(T\right)$, we denote by $E(T,f_{i})$ the set of values of the attribute $f_{i}$ in the table *T*.

A system of equations over *T* is an arbitrary equation system of the following kind:

$$\{f_{i_{1}}=\delta_{1},\ldots ,f_{i_{m}}=\delta_{m}\},$$

where m∈ω, $f_{i_{1}},\ldots ,f_{i_{m}}\in F\left(T\right)$, and $\delta_{1}\in E\left(T,f_{i_{1}}\right),\ldots ,\delta_{m}\in E\left(T,f_{i_{m}}\right)$ (if m=0, then the considered equation system is empty).

Let *T* be a nonempty table. A subtable of *T* is a table obtained from *T* by the removal of some rows. We correspond to each equation system *S* over *T* a subtable TS of the table *T*. If the system *S* is empty, then TS=T. Let *S* be nonempty and S=$\left\{f_{i_{1}}=\delta_{1},\ldots ,f_{i_{m}}=\delta_{m}\right\}$. Then TS is the subtable of the table *T* containing the rows from *T*, which in the intersection with the columns $f_{i_{1}},\ldots ,f_{i_{m}}$ have numbers $\delta_{1},\ldots ,\delta_{m}$, respectively.

## 3. Decision Trees

Let *T* be a nonempty decision table with *n* conditional attributes $f_{1},\ldots ,f_{n}$. We consider the decision trees with two types of queries. We can choose an attribute $f_{i}\in F\left(T\right)=\left\{f_{1},\ldots ,f_{n}\right\}$ and ask about its value. This query has the set of answers $A\left(f_{i}\right)=\left\{\left\{f_{i}=\delta \right\}:\delta \in E\left(T,f_{i}\right)\right\}$. We can formulate a hypothesis over *T* in the form of $H=\{f_{1}=\delta_{1},\ldots ,f_{n}=\delta_{n}\}$, where $\delta_{1}\in E\left(T,f_{1}\right),\ldots ,\delta_{n}\in E\left(T,f_{n}\right)$, and ask about this hypothesis. This query has the set of answers $A\left(H\right)=\{H,\left\{f_{1}=\sigma_{1}\right\},\cdots ,\left\{f_{n}=\sigma_{n}\right\}:\sigma_{1}\in E\left(T,f_{1}\right)\backslash \left\{\delta_{1}\right\},\cdots ,\sigma_{n}\in E\left(T,f_{n}\right)\backslash \left\{\delta_{n}\right\}\}$. The answer *H* means that the hypothesis is true. Other answers are counterexamples. The hypothesis *H* is called proper for *T* if $\left(\delta_{1},\ldots ,\delta_{n}\right)$ is a row of the table *T*.

A decision tree over *T* is a marked finite directed tree with the root in which the following is true:

- Each terminal node is labeled with a number from the set D(T)∪{0}.
- Each node, which is not terminal (such nodes are called working), is labeled with an attribute from the set F(T) or with a hypothesis over *T*.
- If a working node is labeled with an attribute $f_{i}$ from F(T), then for each answer from the set $A(f_{i})$, there is exactly one edge labeled with this answer, which leaves this node and there are no any other edges that leave this node.
- If a working node is labeled with a hypothesis $H=\{f_{1}=\delta_{1},\ldots ,f_{n}=\delta_{n}\}$ over *T*, then for each answer from the set A(H), there is exactly one edge labeled with this answer, which leaves this node and there are no any other edges that leave this node.

Let Γ be a decision tree over *T* and *v* be a node of Γ. We now define an equation system S(Γ,v) over *T* associated with the node *v*. We denote by ξ the directed path from the root of Γ to the node *v*. If there are no working nodes in ξ, then S(Γ,v) is the empty system. Otherwise, S(Γ,v) is the union of equation systems attached to the edges of the path ξ.

A decision tree Γ over *T* is called a decision tree for *T* if for any node *v* of Γ, the following is true:

- The node *v* is terminal if and only if the subtable TS(Γ,v) is degenerate.
- If *v* is a terminal node and the subtable TS(Γ,v) is empty, then the node *v* is labeled with the decision 0.
- If *v* is a terminal node and the subtable TS(Γ,v) is nonempty, then the node *v* is labeled with the decision attached to all rows of TS(Γ,v).

A complete path in Γ is an arbitrary directed path from the root to a terminal node in Γ. As the time complexity of a decision tree, we consider its depth, which is the maximum number of working nodes in a complete path in the tree, or which is the same—the maximum length of a complete path in the tree. We denote by h(Γ) the depth of the decision tree Γ.

As the space complexity of the decision tree Γ, we consider the number of its realizable relative to *T* nodes. A node *v* of Γ is called realizable relative to *T* if and only if the subtable TS(Γ,v) is nonempty. We denote by L(T,Γ) the number of nodes in Γ that are realizable relative to *T*.

In this paper, we consider the following five types of decision trees:

1. Decision trees that use only attributes.
2. Decision trees that use only hypotheses.
3. Decision trees that use both attributes and hypotheses.
4. Decision trees that use only proper hypotheses.
5. Decision trees that use both attributes and proper hypotheses.

## 4. Greedy Algorithm Based on Entropy

Let *T* be a nonempty decision table with *n* conditional attributes $f_{1},\ldots ,f_{n}$ and Θ be a subtable of the table *T*. We define entropy of Θ (denoted ent(Θ)) as follows. If Θ is empty, then ent(Θ)=0. Let Θ be nonempty. For any decision t∈D(Θ), we denote by $N_{t}\left(\Theta \right)$ the number of rows in Θ labeled with the decision *t* and by $p_{t}$ the value $N_{t}\left(\Theta \right)/N\left(\Theta \right)$, where N(Θ) is the number of rows in Θ. Then,

$$ent\left(\Theta \right)=-\sum_{t\in D(T)}p_{t}\log_{2}p_{t}.$$

We now define the impurity of a query for the table Θ. The impurity of the query based on an attribute $f_{i}\in F\left(T\right)$ (impurity of query $f_{i}$) is equal to $I\left(f_{i},\Theta \right)=\max\left\{ent\left(\Theta S\right):S\in A\left(f_{i}\right)\right\}$. The impurity of the query based on a hypothesis *H* over *T* (impurity of query *H*) is equal to I(H,Θ)=max{ent(ΘS):S∈A(H)}.

We can find by simple search among all attributes from F(T) an attribute $f_{i}$ with the minimum impurity $I(f_{i},\Theta )$. We can also find by simple search among all proper hypotheses over *T* a proper hypothesis *H* with the minimum impurity I(H,Θ). It is not necessary to consider all hypotheses over *T* to find a hypothesis with the minimum impurity. For i=1,…,n, we denote by $\delta_{i}$ a number from $E(T,f_{i})$ such that $ent\left(\Theta \left\{f_{i}=\delta_{i}\right\}\right)=\max\left\{ent\left(\Theta \left\{f_{i}=\sigma \right\}\right):\sigma \in E\left(T,f_{i}\right)\right\}$. Then, the hypothesis $H=\{f_{1}=\delta_{1},\ldots ,f_{n}=\delta_{n}\}$ has the minimum impurity I(H,Θ) among all hypotheses over *T*.

We now describe a greedy algorithm E based on entropy that, for a given nonempty decision table *T* and k∈{1,…,5}, constructs a decision tree of the type *k* for the table *T*.

The considered algorithm is similar to standard top-down induction of decision trees [20,21]. The main peculiarity of this algorithm is step 5 in which, depending on the value of *k*, we choose an appropriate set of queries. Every time, the algorithm chooses a query from this set with the minimum impurity.

For a given nonempty decision table *T* and number k∈{1,…,5}, the algorithm 1 E constructs a decision tree of the type *k* for the table *T*. We denote by $h_{E}^{\left(k\right)}\left(T\right)$ the depth of this decision tree and by $L_{E}^{\left(k\right)}\left(T\right)$, we denote the number of realizable relative to *T* nodes in this tree.
**Algorithm 1**E.*Input*: A nonempty decision table *T* and a number k∈{1,…,5}. *Output*: A decision tree of the type *k* for the table *T*.
Construct a tree *G* consisting of a single node labeled with *T*.If no node of the tree *G* is labeled with a table, then the algorithm ends and returns the tree *G*.Choose a node *v* in *G*, which is labeled with a subtable Θ of the table *T*.If Θ is degenerate, then instead of Θ, we label the node *v* with 0 if Θ is empty and with the decision attached to each row of Θ if Θ is nonempty.If Θ is nondegenerate, then depending on *k*, we choose a query *X* (either attribute or hypothesis) in the following way:(a)If k=1, then we find an attribute *X*∈F(T) with the minimum impurity I(X,Θ).(b)If k=2, then we find a hypothesis *X* over *T* with the minimum impurity I(X,Θ).(c)If k=3, then we find an attribute *Y*∈F(T) with the minimum impurity I(Y,Θ) and a hypothesis *Z* over *T* with the minimum impurity I(Z,Θ). Between *Y* and *Z*, we choose a query *X* with the minimum impurity I(X,Θ).(d)If k=4, then we find a proper hypothesis *X* over *T* with the minimum impurity I(X,Θ).(e)If k=5, then we find an attribute *Y*∈F(T) with the minimum impurity I(Y,Θ) and a proper hypothesis *Z* over *T* with the minimum impurity I(Z,Θ). Between *Y* and *Z*, we choose a query *X* with the minimum impurity I(X,Θ).Instead of Θ, we label the node *v* with the query *X*. For each answer S∈A(X), we add to the tree *G* a node v(S) and an edge e(S) connecting *v* and v(S). We label the node v(S) with the subtable ΘS and label the edge e(S) with the answer *S*. We then proceed to step 2.


## 5. Results of Experiments

We make experiments with 10 decision tables from the UCI ML Repository [19]. Table 1 contains the information about each of these decision tables: its name, the number of rows, and the number of conditional attributes.

The results of the experiments are represented in Table 2 and Table 3. The first column of Table 2 contains the name of the considered decision table *T*. The last five columns contain values $h_{E}^{\left(1\right)}\left(T\right),\ldots ,h_{E}^{\left(5\right)}\left(T\right)$ (minimum values for each decision table are in bold).

The first column of Table 3 contains the name of the considered decision table *T*. The last five columns contain values $L_{E}^{\left(1\right)}\left(T\right),\ldots ,L_{E}^{\left(5\right)}\left(T\right)$ (minimum values for each decision table are in bold).

For n=3,…,6, we randomly generate 100 Boolean functions with *n* variables. We represent each Boolean function with *n* variables as a decision table with *n* columns labeled with these variables and with $2^{n}$ rows that are all possible *n*-tuples of values of the variables. Each row is labeled with the decision that is the value of the function on the corresponding *n*-tuple.

For each function, using its decision table representation and the algorithm E, we construct a decision tree of the type *k* computing this function, k=1,…,5. For each Boolean function, each hypothesis over the decision table representing it is proper. Therefore, for each Boolean function, $h_{E}^{\left(2\right)}=h_{E}^{\left(4\right)}$, $h_{E}^{\left(3\right)}=h_{E}^{\left(5\right)}$, $L_{E}^{\left(2\right)}=L_{E}^{\left(4\right)}$, and $L_{E}^{\left(3\right)}=L_{E}^{\left(5\right)}$.

The results of the experiments are represented in Table 4 and Table 5. The first column in Table 4 contains the number of variables *n* in the considered Boolean functions. The last five columns contain information about values $h_{E}^{\left(1\right)},\ldots ,h_{E}^{\left(5\right)}$ in the format ${}_{\min}{\mathrm{Avg}}_{\max}$.

The first column in Table 5 contains the number of variables *n* in the considered Boolean functions. The last five columns contain information about values $L_{E}^{\left(1\right)},\ldots ,L_{E}^{\left(5\right)}$ in the format ${}_{\min}{\mathrm{Avg}}_{\max}$.

From the obtained experimental results, it follows that, using hypotheses, we can decrease the depth of the constructed decision trees. However, at the same time, the number of realizable nodes usually grows. Depending on our goals, we should choose decision trees of type 3 if we would like to minimize the depth and decision trees of type 1 if we would like to minimize the number of realizable nodes.

## 6. Conclusions

In this paper, we studied modified decision trees that use both queries based on one attribute each and queries based on hypotheses about the values of all attributes. We proposed an entropy-based greedy algorithm for the construction of such decision trees and considered the results of computer experiments. The goal of this paper is to understand which type of decision tree should be chosen if we would like to minimize the depth and which type should be chosen if we would like to minimize the number of realizable nodes. From the obtained experimental results, it follows that we should choose decision trees of type 3 if we would like to minimize the depth and decision trees of type 1 if we would like to minimize the number of realizable nodes. The comparison of different greedy algorithms based on various uncertainty measures will be done in future papers.

## Figures and Tables

**Table 1:** Decision tables from [19] used in experiments.

Decision	Number of	Number of
Table	Rows	Attributes
balance-scale	625	5
breast-cancer	266	10
cars	1728	7
hayes-roth-data	69	5
lymphography	148	18
nursery	12,960	9
soybean-small	47	36
spect-test	169	22
tic-tac-toe	958	10
zoo-data	59	17

**Table 2:** Experimental results for decision tables from [19] (depth).

Decision Table *T*	$h_{E}^{\left(1\right)}\left(T\right)$	$h_{E}^{\left(2\right)}\left(T\right)$	$h_{E}^{\left(3\right)}\left(T\right)$	$h_{E}^{\left(4\right)}\left(T\right)$	$h_{E}^{\left(5\right)}\left(T\right)$
balance-scale	**4**	**4**	**4**	**4**	**4**
breast-cancer	9	9	9	**8**	9
cars	**6**	**6**	**6**	**6**	**6**
hayes-roth-data	**4**	**4**	**4**	**4**	**4**
lymphography	11	11	**9**	13	10
nursery	**8**	**8**	**8**	**8**	**8**
soybean-small	**2**	6	**2**	8	**2**
spect-test	20	**5**	**5**	14	11
tic-tac-toe	**7**	8	**7**	8	**7**
zoo-data	8	6	**5**	8	**5**
Average	7.9	6.7	5.9	8.1	6.6

**Table 3:** Experimental results for decision tables from [19] (number of realizable nodes).

Decision Table *T*	$L_{E}^{\left(1\right)}\left(T\right)$	$L_{E}^{\left(2\right)}\left(T\right)$	$L_{E}^{\left(3\right)}\left(T\right)$	$L_{E}^{\left(4\right)}\left(T\right)$	$L_{E}^{\left(5\right)}\left(T\right)$
balance-scale	**556**	5234	4102	5234	4102
breast-cancer	**255**	446,170	304	10,3642	266
cars	**1136**	65,624	3944	65,624	3944
hayes-roth-data	73	**72**	**72**	367	**72**
lymphography	**123**	6,653,366	162	8,515,841	153
nursery	**4460**	12,790,306	14,422	12,790,306	14,422
soybean-small	**7**	10,029	**7**	157,640	**7**
spect-test	**123**	6983	5495	398,926	1116
tic-tac-toe	**648**	864,578	200,847	946,858	940
zoo-data	**33**	2134	35	13,310	35
Average	741.4	2,084,449.6	22,939	2,299,774.8	2505.7

**Table 4:** Experimental results for Boolean functions (depth).

Number of Variables *n*	$h_{E}^{\left(1\right)}$	$h_{E}^{\left(2\right)}$	$h_{E}^{\left(3\right)}$	$h_{E}^{\left(4\right)}$	$h_{E}^{\left(5\right)}$
3	${}_{0}2.94_{3}$	${}_{0}2.02_{3}$	${}_{0}1.86_{3}$	${}_{0}2.02_{3}$	${}_{0}1.86_{3}$
4	${}_{4}4.00_{4}$	${}_{2}3.05_{4}$	${}_{2}2.97_{3}$	${}_{2}3.05_{4}$	${}_{2}2.97_{3}$
5	${}_{5}5.00_{5}$	${}_{4}4.11_{5}$	${}_{3}3.99_{4}$	${}_{4}4.11_{5}$	${}_{3}3.99_{4}$
6	${}_{6}6.00_{6}$	${}_{5}5.09_{6}$	${}_{5}5.00_{5}$	${}_{5}5.09_{6}$	${}_{5}5.00_{5}$

**Table 5:** Experimental results for Boolean functions (number of realizable nodes).

Number of Variables *n*	$L_{E}^{\left(1\right)}$	$L_{E}^{\left(2\right)}$	$L_{E}^{\left(3\right)}$	$L_{E}^{\left(4\right)}$	$L_{E}^{\left(5\right)}$
3	${}_{1}9.60_{15}$	${}_{1}12.33_{22}$	${}_{1}9.61_{15}$	${}_{1}12.33_{22}$	${}_{1}9.61_{15}$
4	${}_{15}21.02_{29}$	${}_{14}44.75_{70}$	${}_{11}27.69_{58}$	${}_{14}44.75_{70}$	${}_{11}27.69_{58}$
5	${}_{31}42.54_{51}$	${}_{125}218.05_{292}$	${}_{25}70.19_{176}$	${}_{125}218.05_{292}$	${}_{25}70.19_{176}$
6	${}_{69}86.30_{101}$	${}_{649}1171.03_{1538}$	${}_{75}292.99_{807}$	${}_{649}1171.03_{1538}$	${}_{75}292.99_{807}$

## Data Availability

Data available in a publicly accessible repository that does not issue DOIs. Publicly available data sets were analyzed in this study. These data can be found here: http://archive.ics.uci.edu/ml accessed date: 12 April 2017.
